# Predictive effect of the decline in CD4^+^ T cell levels in blood on infection in patients with severe hemorrhagic stroke and mechanism

**DOI:** 10.3389/fneur.2023.1118282

**Published:** 2023-06-09

**Authors:** Yating Wang, Junshuang Guo, Fan Yang, Ruirui Dong, Dandan Song, Peipei Huang, Lijun Wen, Guoliang Xiang, Shuiyu Wang, Junfang Teng, Wang Miao

**Affiliations:** ^1^Neuro-Intensive Care Unit of the First Affiliated Hospital of Zhengzhou University, Zhengzhou, Henan, China; ^2^Department of Immunology, School of Basic Medical Science, Central South University, Changsha, Hunan, China

**Keywords:** severe hemorrhagic stroke, infection, CD4^+^ T cells, IL-6, IL-8

## Abstract

**Objective:**

The purpose of this research was to evaluate the influence of immunity on infection in patients with severe hemorrhagic stroke and explore the mechanism underlying this connection.

**Methods:**

Clinical data obtained from 126 patients with severe hemorrhagic stroke were retrospectively analyzed, and the factors affecting infection were screened by multivariable logistic regression models. Nomograms, calibration curves, the Hosmer–Lemeshow goodness-of-fit test, and decision curve analysis were used to examine the effectiveness of the models in evaluating infection. The mechanism underlying the reduction in CD4^+^ T-cell levels in blood was explored by analysis of lymphocyte subsets and cytokines in cerebrospinal fluid (CSF) and blood.

**Results:**

The results showed that CD4^+^ T-cell levels of <300/μL was an independent risk factor for early infection. The models for multivariable logistic regression involving the CD4^+^ T-cell levels and other influencing factors had good applicability and effectiveness in evaluating early infection. CD4^+^ T-cell levels decreased in blood but increased in CSF. Similarly, interleukin (IL)-6 and IL-8 levels in CSF had a significant increase, generating a substantial concentration gradient between the CSF and the blood.

**Conclusion:**

Reduced blood CD4^+^ T-cell counts among patients who had severe hemorrhagic stroke increased the risk of early infection. CSF IL-6 and IL-8 may be involved in inducing the migration of CD4^+^ T cells into the CSF and decreasing blood CD4^+^ T-cell levels.

## 1. Introduction

Stroke affects human life expectancy and is the second most prominent cause of death (1). Hemorrhagic stroke, especially severe hemorrhagic stroke, has a high mortality and disability rate and is an important contributor to stroke mortality (2). Post-stroke infection has been found to increase the rate of death and disability in patients (3), so the prevention and treatment of post-stroke infection have become important measures to reduce the rate of death and disability due to hemorrhagic stroke.

In patients with severe hemorrhagic stroke, intense stress causes immunosuppression (4) and decreased metabolism (5), and consciousness disorder is severe. Obvious disorders of the immune system and barrier function destruction cause immunosuppression, reducing the body's ability to fight against bacteria (4). Low triiodothyronine syndrome occurs during stressful states of critical illness (5, 6), resulting in decreased metabolism, which increases the risk of infection (7), although initially, this process is a protective measure (8). Severe hemorrhagic stroke patients often present with consciousness disorders and usually need mechanical ventilation as well as admission to the intensive care unit (ICU). The incidence of infection and even multi-drug resistant organism infection in such patients is higher (9, 10).

CD4^+^ T cells are the key cells involved in adaptive immunity (11, 12), and their levels have great value in judging the strength of the body's immunity. Less than 300/μL and 200/μL are the usual cut-off values defined for CD4^+^ T-cell reduction (13, 14), reflecting varying degrees of immunosuppression. However, the relationship between immunosuppression and infection in patients with severe hemorrhagic stroke is not clear. Moreover, CD4^+^ T cells can access sites of inflammation in the central nervous system due to cytokine chemotaxis (15), which may be relevant to the pathological mechanisms of hemorrhagic stroke.

Infection early after stroke is frequent (16), and a previous study has shown that the infection occurrence rate after a stroke is 5–65% (17). The occurrence of infection not only prolongs the patient's hospital stay and increases the cost (18) but also reduces the prognosis and increases the mortality rate (3), which is the main challenge currently faced by critically ill patients. Therefore, identifying the relevant predictors of infection in patients with severe hemorrhagic stroke and taking measures to reduce infection occurrence will help to improve the prognosis of patients and reduce mortality. However, there has been limited analysis of the factors influencing early infection in patients with severe hemorrhagic stroke, especially regarding immunity, which were explored in this study.

## 2. Methods

### 2.1. Research participants

**Figure 1:**
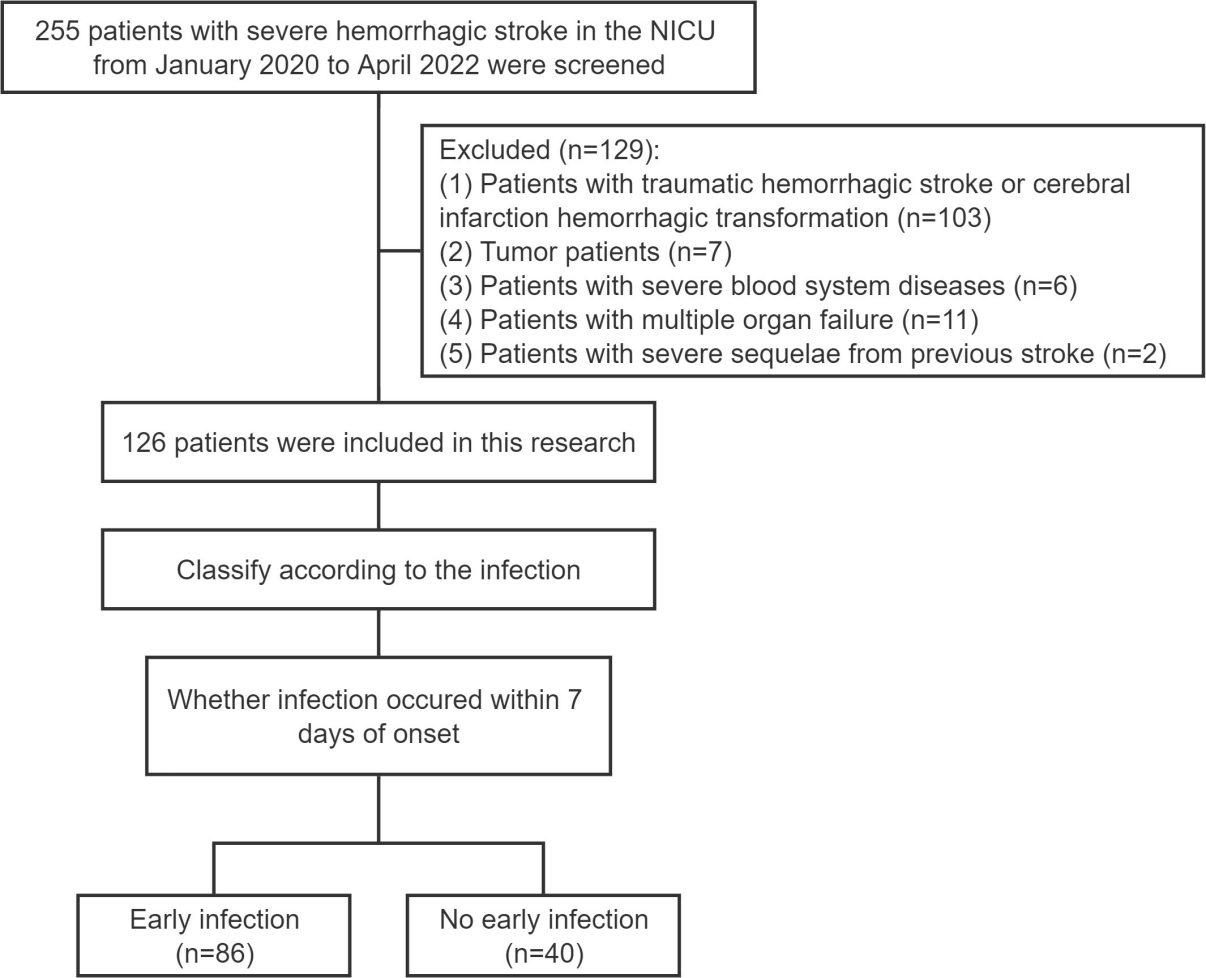
The selection process used for the study population. NICU, neurointensive care unit.

Two hundred and fifty five patients with severe hemorrhagic stroke in the neurointensive care unit (NICU) of the First Affiliated Hospital of Zhengzhou University from January 2020 to April 2022 were screened. The criteria for inclusion were as follows: (1) patients with spontaneous hemorrhagic stroke within 3 days of onset, which was confirmed by head CT scan; (2) patients requiring close monitoring such as cerebral herniation, coma, or assisted ventilation due to the hemorrhagic event; (3) patients who were not co-infected on admission and who were not receiving antiviral, antibiotic or other interventional therapy; (4) patients between 18 and 80 years old; (5) patients whose hospital length of stay (LOS) was more than or equal to 7 days; and (6) patients who had complete clinical data and laboratory test results. The following were the exclusion criteria: (1) patients with traumatic hemorrhagic stroke or cerebral infarction hemorrhagic transformation (103 patients); (2) tumor patients (7 patients); (3) patients with severe blood system diseases (6 patients); (4) patients with multiple organ failure (11 patients); and (5) patients with severe sequelae from previous stroke (2 patients). As detailed in Figure 1, 126 patients were included in this research. This study was reviewed and approved by the ethics committee of the First Affiliated Hospital of Zhengzhou University (approval no. 2022-KY-0801-001).

## 3. Research methods

Clinical characteristics and auxiliary findings included temperature at admission, systolic and diastolic blood pressure on admission, symptoms, comorbidities, routine blood analysis, flow cytometry for analysis of lymphocyte subsets, cytokine analysis, and thyroid function analysis. Routine blood analyses were measured on the day of admission for 126 patients, lymphocyte subsets and cytokine analysis and thyroid function were tested the following morning. The lymphocyte subsets and cytokine samples from the cerebrospinal fluid (CSF) and blood of the 11 patients in Part II were tested on the same day as the initial lumbar puncture (within 3 days of admission). Less than 2% of patients had missing results for lymphocyte subsets and cytokine assays in their blood, which were filled in with the mean of the included patients. Follow-up data included ICU LOS, hospital LOS, and 3-month prognosis.

In this study, early infection referred to an infection that occurred within 7 days of the onset of stroke symptoms (16). Infection was determined by the treating physician using the guidelines of the Centers for Disease Control and Prevention criteria (19, 20).

The prognosis was evaluated by the modified Rankin score (mRS) performed 3 months after stroke. The definition of a good prognosis is an mRS score of 0 to 3, and a poor prognosis is an mRS score of 4 to 5 or death (mRS score of 6).

### 3.1. Statistical analysis

The data for continuous variables that conformed to a normal distribution are displayed as the mean ± standard deviation (SD) and were compared using the *t*-test. The Mann–Whitney U test was applied to compare non-normally distributed data, which are shown as the median (IQR: interquartile range). To compare categorical data, which involved case counts (percentage), a chi-square test was performed. The variables that had a significant difference in single-factor analysis were incorporated into multivariable regression analysis. Nomograms were created under the outcomes of multivariable logistic regression. Bootstrapping was utilized for internal validation. To assess the model's predictive power and accuracy, calibration curves, the Hosmer–Lemeshow goodness-of-fit test, and decision curve analysis were established. Log-rank test testing was performed on Kaplan–Meier survival curves. P < 0.05 was chosen to define the statistical significance level. Statistical analysis was completed by using SPSS 21.0, GraphPad Prism 8, and R software.

## 4. Results

### 4.1. Part I: decreased blood CD4^+^ T-cell count in patients with severe hemorrhagic stroke increases the risk for early infection

#### 4.1.1. Infection in severe hemorrhagic stroke patients

A total of 126 patients with severe hemorrhagic stroke participated in this research, with a mean age of 55.98 years (SD 12.19), including 73 males (57.9%) and 53 females (42.1%) (Table 1).

**Table 1:** Comparison of clinical data between the early infection group and the group without early infection.

**Variable**	**Total (*n* = 126)**	**Early infection (*n* = 86)**	**No early infection (*n* = 40)**	***P–*value**
Male, *n* (%)	73 (57.9)	46 (53.5)	27 (67.5)	0.138
Age, mean ± SD, year	55.98 ± 12.19	56.23 ± 12.76	55.43 ± 11.00	0.731
Temperature, median (IQR), °C	36.90 (36.70–37.10)	37.00 (36.70–37.20)	36.90 (36.80–37.00)	0.918
SBP, median (IQR), mmHg	157.00 (140.00–177.00)	158.00 (140.00–174.25)	157.00 (140.25–177.75)	0.620
DBP, median (IQR), mmHg	91.00 (78.00–103.25)	91.00 (78.00–102.00)	90.50 (81.50–104.75)	0.432
**Symptoms**				
Consciousness disorder ^a^, *n* (%)	79 (62.7)	68 (79.1)	11 (27.5)	< 0.001
Limb weakness, *n* (%)	63 (50.0)	45 (52.3)	18 (45.0)	0.444
Dizziness/headache, *n* (%)	76 (60.3)	50 (58.1)	26 (65.0)	0.464
Slurred speech, *n* (%)	36 (28.6)	23 (26.7)	13 (32.5)	0.506
Visual impairment, *n* (%)	6 (4.8)	2 (2.3)	4 (10.0)	0.152
Mouth askew/salivation, *n* (%)	11 (8.7)	8 (9.3)	3 (7.5)	1.000
Ataxia, *n* (%)	3 (2.4)	2 (2.3)	1 (2.5)	1.000
Fecal or urinary incontinence, *n* (%)	11 (8.7)	10 (11.6)	1 (2.5)	0.177
Body convulsions, *n* (%)	7 (5.6)	4 (4.7)	3 (7.5)	0.816
Days from symptom onset to admission, median (IQR)	0.44 (0.25–1.28)	0.42 (0.21–1.02)	0.75 (0.25–2.00)	0.109
**Comorbidities**				
Hypertension, *n* (%)	80 (63.5)	52 (60.5)	28 (70.0)	0.301
Diabetes, *n* (%)	13 (10.3)	12 (14.0)	1 (2.5)	0.098
**Blood exams**				
WBC, median (IQR), × 10^9^/L	11.43 (8.66–14.14)	12.47 (9.13–14.52)	9.04 (7.54–11.27)	< 0.001
Neutrophil, median (IQR), × 10^9^/L	10.22 (7.25–12.68)	11.25 (7.86–13.06)	7.66 (6.63–10.29)	< 0.001
Lymphocyte, median (IQR), × 10^9^/L	0.69 (0.55–1.00)	0.68 (0.49–1.01)	0.79 (0.59–1.00)	0.214
B cells, median (IQR),/μL	148.65 (88.48–224.37)	152.15 (81.79–219.03)	132.41 (98.51–233.80)	0.801
CD4^+^ T cells, median (IQR),/μL	308.41 (206.79–485.83)	268.67 (195.16–454.97)	360.05 (253.86–569.50)	0.009
CD8^+^ T cells, median (IQR),/μL	182.56 (112.22–298.35)	170.57 (109.65–294.34)	191.29 (136.66–342.84)	0.238
NK cells, median (IQR),/μL	144.65 (86.58–192.97)	140.69 (84.45–194.29)	157.04 (91.33–193.31)	0.675
CD4^+^ T cells <300/μL, *n* (%)	61 (48.4)	49 (57.0)	12 (30.0)	0.005
CD4^+^ T cells <200/μL, *n* (%)	27 (21.4)	22 (25.6)	5 (12.5)	0.096
IL−1β, median (IQR), pg/mL	2.12 (1.04–3.06)	2.05 (0.95–2.87)	2.52 (1.21–3.78)	0.197
IL−6, median (IQR), pg/mL	11.21 (5.68–41.64)	20.80 (6.50–53.64)	6.77 (3.13–11.91)	< 0.001
IL−8, median (IQR), pg/mL	7.38 (4.79–12.44)	8.18 (5.30–13.99)	6.48 (4.05–9.91)	0.013
IL−10, median (IQR), pg/mL	3.91 (2.28–6.29)	3.53 (1.93–6.17)	4.41 (2.77–7.30)	0.277
IFN–γ, median (IQR), pg/mL	1.25 (0.80–1.61)	1.16 (0.80–1.62)	1.38 (0.95–1.62)	0.229
FT3, mean ± SD, pmol/L	4.19 ± 0.74	4.12 ± 0.77	4.34 ± 0.65	0.115
FT4, median (IQR), pmol/L	11.57 (10.31–13.08)	11.77 (10.28–13.38)	11.36 (10.37–12.60)	0.408
TSH, median (IQR), μIU/mL	0.55 (0.34–0.97)	0.56 (0.32,0.97)	0.55 (0.38, 0.97)	0.854

SD, standard deviation; IQR, interquartile range; SBP, systolic blood pressure; DBP, diastolic blood pressure; WBC, white blood cell; NK cells: natural killer cells; IL-1β, interleukin-1beta; IL-6, interleukin-6; IL-8, interleukin-8; IL-10, interleukin-10; IFN-γ, interferon gamma; FT3, free triiodothyronine; FT4, free thyroxine; TSH, thyroid stimulating hormone.

a , Glasgow Coma Scale ≤ 12 points.

Among them, infection in 86 (68%) patients occurred within 7 days of stroke (early infection), and 40 (32%) patients did not develop early infection (Table 1). Of the 86 patients with early infection, 81 patients had a lung infection, 1 patient developed an infection of the urinary tract, and 4 had both a lung and urinary tract infection.

#### 4.1.2. Effect of infection in patients with severe hemorrhagic stroke on ICU LOS, hospital LOS, and prognosis

ICU LOS was longer for patients in the early infection group than for those in the group without early infection (*P* < 0.001), and hospital LOS was not significantly different between the two groups (*P* > 0.05) (Table 2). The prognosis of 118 patients was able to be followed up at 3 months (eight patients could not be followed up due to a change of contact details). When patients experienced complications with early infection, the mRS prognostic score assessed during the 3-month follow-up was poor (*P* < 0.001) (Table 2).

**Table 2:** Comparison of follow-up data between the early infection group and the group without early infection.

**LOS**	**Total (*n* = 126)**	**Early infection (*n* = 86)**	**No early infection (*n* = 40)**	***P-*value**
ICU LOS, median (IQR)	11.50 (7.00–19.25)	14.00 (8.00–22.00)	7.00 (4.00–10.75)	< 0.001
Hospital LOS, median (IQR)	21.50 (15.00–33.00)	22.00 (15.00–34.00)	20.50 (15.00–32.00)	0.548
**Prognosis**	**Total (*****n*** = **118)**	**Early infection (*****n*** = **79)**	**No early infection (*****n*** = **39)**	***P-*** **value**
mRS after 3 months ≤ 3, *n* (%)	68 (57.6)	35 (44.3)	33 (84.6)	< 0.001

LOS, length of stay; ICU, intensive care unit; IQR, interquartile range; mRS, modified Rankin score.

#### 4.1.3. Comparison of clinical data between the early infection group and the group that did not exhibit early infection

Patients with early infection had a higher incidence of consciousness disorder (*P* < 0.001), higher white blood cell (WBC) levels (*P* < 0.001), and higher neutrophil counts (*P* < 0.001) than patients without early infection. The early infection group had more patients with CD4^+^ T-cell counts < 300/μL (*P* = 0.005), as well as higher interleukin (IL)-6 (*P* < 0.001) and IL-8 (*P* = 0.013) levels. There were significant differences between the two groups (*P* < 0.05) (Table 1).

As shown in Table 1, the median CD4^+^ T-cell count in 126 patients with severe hemorrhagic stroke was 308.41/μL (IQR 206.79/μL–485.83/μL), which was less than the normal range (550–1,440/μL). This study used the common clinical cut-off values of 300 and 200 to convert CD4^+^ T-cell counts into dichotomous variables, representing different degrees of immunosuppression and enabling more instructive clinical diagnosis and treatment.

#### 4.1.4. Multivariable logistic regression for early infection

**Figure 2:**
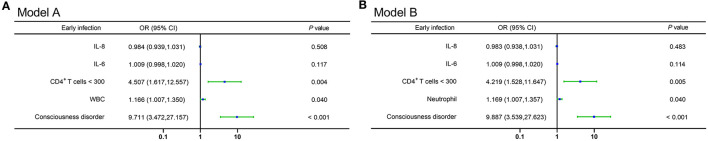
**(A, B)** Forest plots of multivariable logistic regression affecting early infection in patients (Model A and Model B). OR, odds ratio; CI, confidence interval; IL-8, interleukin-8; IL-6, interleukin-6; WBC, white blood cell.

To identify the influencing factors for early infection in severe hemorrhagic stroke patients, the significantly different factors shown in Table 1 were incorporated in a multivariable logistic regression model. Because the variance inflation factors of WBC counts and neutrophil levels were >10, suggesting the presence of multicollinearity, they were separated and included in two models (Figures 2A, B). The results showed that consciousness disorder, WBC count, neutrophil levels, and CD4^+^ T-cell counts <300/μL were independent risk elements of early infection in patients.

#### 4.1.5. Nomograms, calibration curves, and decision curve analysis

**Figure 3:**
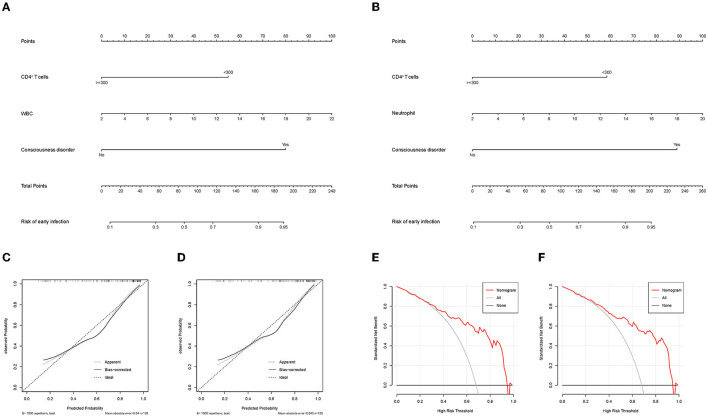
**(A)** Nomogram that includes CD4^+^ T cell levels, WBC levels, and consciousness disorder for assessing early infection in patients. **(B)** Nomogram that includes CD4^+^ T cell levels, neutrophil counts, and consciousness disorder for assessing early infection in patients. **(C, D)** The calibration curve that corresponds to the nomogram; the observation curve is close to 45°. **(E, F)** Decision curve analysis for assessing early infection. **(C, E)** correspond to **(A)**. **(D, F)** correspond to **(B)**. WBC, white blood cell.

To assess the risk for early infection in severe hemorrhagic stroke patients, nomograms based on multivariable logistic regression were generated, and using bootstrapping for validation, the c-index values were 0.853 (95% CI: 0.783–0.924) (Figure 3A) and 0.851 (95% CI: 0.779–0.922) (Figure 3B) in each model. The models of multivariable logistic regression that involved CD4^+^ T lymphocyte levels (CD4^+^ T-cell count <300/μL) had great assessment efficacy. Moreover, the calibration curves showed a good fit during internal validation (Figures 3C, D), and the Hosmer–Lemeshow goodness-of-fit test showed that the predicted values in the model approached the observed values (χ^2^ = 5.045, *p* = 0.753. χ^2^ = 6.624, *p* = 0.578). Evaluation of the applicability and validity of the nomogram plots by decision curve analysis showed a net clinical benefit of our model (Figures 3E, F).

### 4.2. Part II: analysis of the causes and effects of the decrease in blood CD4^+^ T-cell levels in patients with severe hemorrhagic stroke

#### 4.2.1. Elevated cytokines in the CSF in patients with severe hemorrhagic stroke may be responsible for inducing the migration of CD4^+^ T cells into the CSF

**Figure 4:**
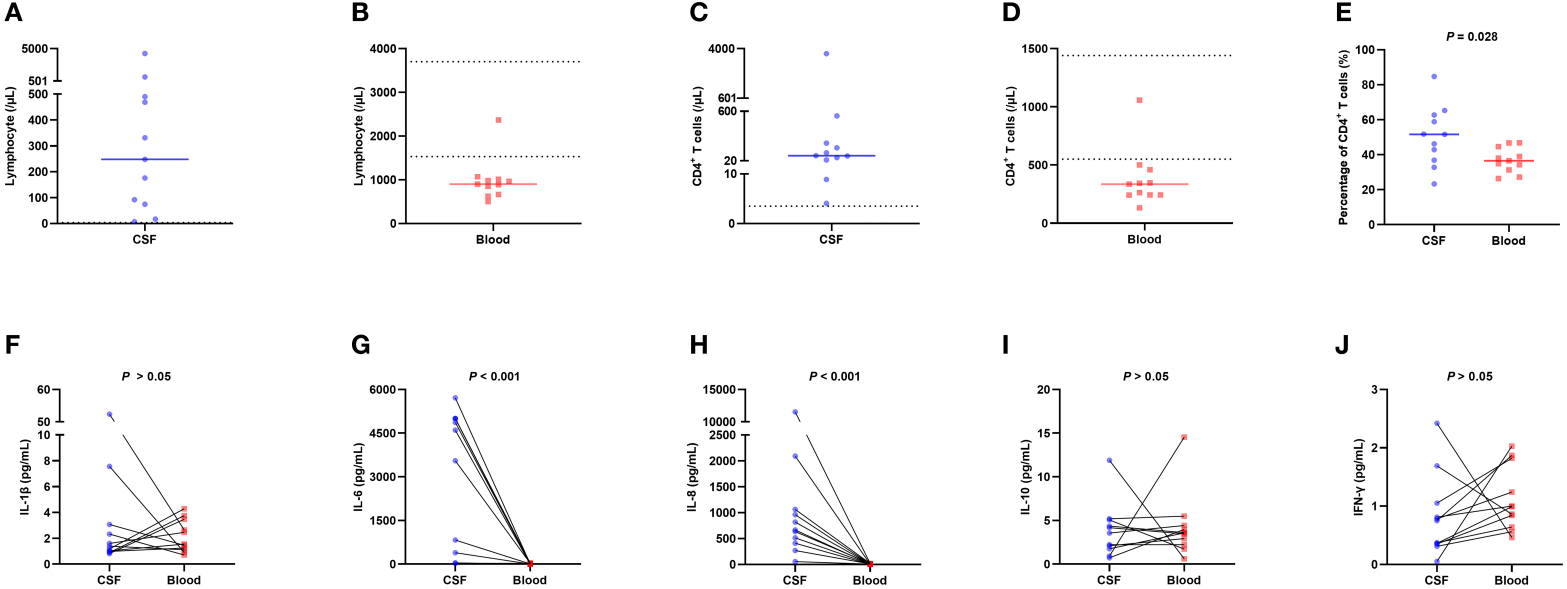
Flow cytometry lymphocyte subset analysis and cytokine measurements in 11 patients with cerebral parenchymal hemorrhage breaking into the ventricular system or subarachnoid space. **(A)** The total number of lymphocytes in CSF, with a median of 248.06/μL (IQR 74.40/μL −490.35/μL), was significantly higher than normal. **(B)** The total number of lymphocytes in blood, with a median of 900.67/μL (IQR 664.01/μL −1011.33/μL), was lower than normal. **(C)** The total number of CD4^+^ T cells in CSF, with a median of 81.38/μL (IQR 31.87/μL −226.46/μL), was significantly higher than normal. **(D)** The total number of CD4^+^ T cells in blood, with a median of 336.21/μL (IQR 242.76/μL −459.03/μL), was lower than normal. **(E)** The percentage of CD4^+^ T cells in CSF was higher than that in blood. **(F–J)** Distribution of IL-1β, IL-6, IL-8, IL-10, and IFN-γ levels in CSF and blood. The results showed that the levels of the cytokines IL-6 and IL-8 in CSF were significantly higher than those in blood (median 4603.09 vs. 8.24, *P* < 0.001; median 665.36 vs. 7.08, *P* < 0.001), while the levels of other cytokines did not differ significantly. The solid line in the ABCDE chart represents the median, and the dashed line in the ABCD chart represents the normal value. CSF, cerebrospinal fluid; IL-1β, interleukin-1beta; IL-6, interleukin-6; IL-8, interleukin-8; IL-10, interleukin-10; IFN-γ, interferon gamma; IQR, interquartile range.

To explore the causes and potential mechanisms in which peripheral blood CD4^+^ T cells are involved in patients who suffered a severe hemorrhagic stroke, we analyzed the lymphocytes subset of CSF and blood in 11 patients with cerebral parenchymal hemorrhage breaking into the ventricular system or subarachnoid space (Figures 4A–E), and the outcomes revealed that the median total number of lymphocytes in CSF was 248.06/μL (IQR 74.40/μL–490.35/μL) (Figure 4A). The normal WBC count in CSF is <5/μL, the normal proportion of lymphocytes is 60%−70%, and the normal level of lymphocytes in CSF is estimated to be 0–3.5/μL, so the total number of lymphocytes in CSF in patients with severe hemorrhagic stroke was significantly higher than normal. The median total number of lymphocytes in the blood was 900.67/μL (IQR 664.01/μL −1011.33/μL) (the normal value is 1,530–3,700/μL) (Figure 4B), which was significantly lower than normal, and the blood and CSF showed the opposite trends. The classification of lymphocyte subsets showed that the CD4^+^ T-cell levels were elevated in CSF, which was also contrary to the decreasing tendency in the blood. The median CD4^+^ T-cell count in CSF was 81.38/μL (IQR 31.87/μL −226.46/μL) (Figure 4C), the median CD4^+^ T-cell count in blood was 336.21/μL (IQR 242.76/μL−459.03/μL) (normal range 550–1,440/μL) (Figure 4D), and the percentage of CD4^+^ T cells in CSF was larger than that in blood (Figure 4E).

To investigate the mechanism underlying the elevation in CD4^+^ T-cell counts in the CSF of patients with severe hemorrhagic stroke, we measured cytokine levels in the CSF and blood obtained from these 11 patients (Figures 4F–J), and the results demonstrated that IL-6 and IL-8 in CSF had significantly higher levels than those in the blood (median 4603.09 vs. 8.24, *P* < 0.001; median 665.36 vs. 7.08, *P* < 0.001), while the levels of other cytokines did not differ significantly.

#### 4.2.2. Effect of CD4^+^ T lymphopenia on the incidence and timing of early infection

The outcomes of multivariable logistic regression demonstrated that CD4^+^ T-cell levels of <300/μL was independent risk factor for early infection. Kaplan–Meier analysis was used to further explore the effect of CD4^+^ T-cell counts of <300/μL on the overall probability of early infection.

**Figure 5:**
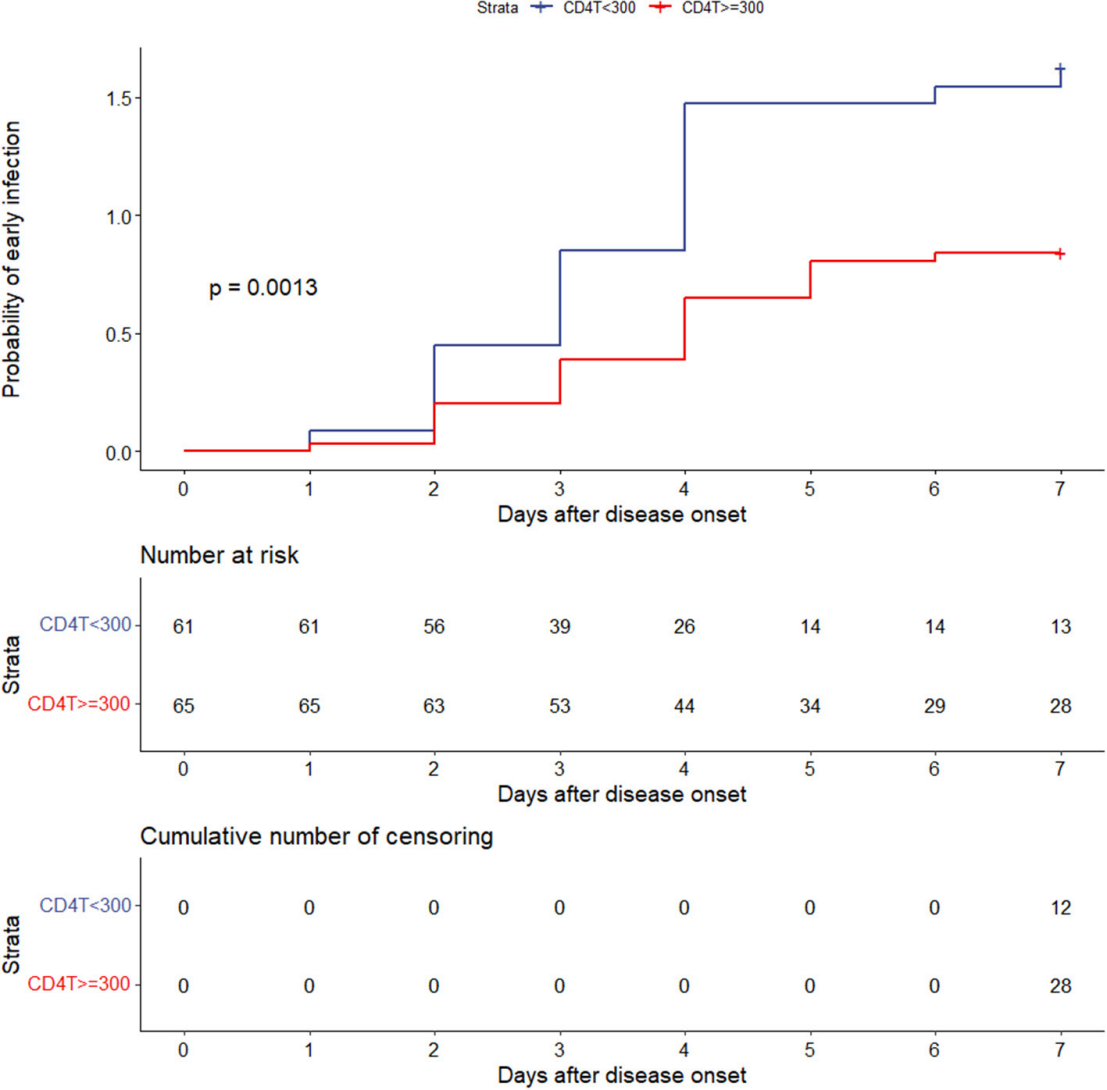
Effect of CD4^+^ T-cell levels of <300 or ≥300/μL on the overall probability of early infection. CD4T, CD4^+^ T cells.

CD4^+^ T-cell levels <300/μL and CD4^+^ T-cell levels ≥ 300/μL were found to have a significantly different effect on the overall probability of having an early infection (*P* = 0.0013) (Figure 5).

## 5. Discussion

According to the findings of this study, the decrease in blood CD4^+^ T-cell levels and the occurrence of consciousness disorder in patients who had severe hemorrhagic stroke during the acute phase had obvious predictive value for the occurrence of early infection. In addition to consciousness disorder increasing the patient's risk of infection, the risk for early infection increases when CD4^+^ T-cell levels in the blood are <300/μL (Figures 2, 3). The presence of infection increases patient ICU LOS and is associated with a worse prognosis. This finding provides insights on clinical prevention and treatment.

In contrast to a decrease in blood CD4^+^ T-cell levels, CD4^+^ T-cell levels in CSF were markedly elevated (Figure 4). CD4^+^ T cells in CSF may originate from CD4^+^ T-cell migration from the blood (21), which may be one of the reasons for the reduction in blood CD4^+^ T-cell levels in patients with severe hemorrhagic stroke. CD4^+^ T cells are vital cells in the human immune system and play an important role in alleviating bacterial and viral infections and in tumor immunity (11, 12).

Severe hemorrhagic stroke is often associated with consciousness disorder, and various reflexes, such as cough, are reduced, which increases the risk of infection.

Infection frequently occurs in patients who suffer from severe hemorrhagic stroke. Our study showed that the rate of early infection in 126 patients with severe hemorrhagic stroke was 68%. Previous research has found that the infection rate is 5–65% in stroke patients (17). The samples included in our study were obtained from patients with severe hemorrhagic stroke who, in addition to being critically ill, often required ventilators to assist breathing, invasive procedures, and the indwelling of various tubes, which may have been responsible for the high infection rate of these patients. Patients with severe hemorrhagic stroke who exhibit complications with infection have a longer ICU LOS and a poorer prognosis than those without infection, which not only increases the treatment period and cost to patients but also brings adverse effects to patients and increases burdens on families and society.

Our results also showed that WBC counts and neutrophil levels increased the risk of early infection to some extent. These cells play an important role in mediating inflammatory responses (22), but in this study, they had less influence on early infection than CD4^+^ T cells and consciousness disorder, and from the perspective of infection causality, they are more suitable as indicators to define infection than to predict infection.

To more comprehensively investigate the value of these indicators during the evaluation of infection, nomograms were generated to assess the risk of early infection, and further evaluation was performed using calibration curves, the Hosmer–Lemeshow goodness-of-fit test, and decision curve analysis. The outcomes showed that the predictions were generally consistent with the actual observations, which could increase the convenience of this analysis for clinicians during the assessment of infection in patients who have severe hemorrhagic stroke.

The purpose of the evaluation is not only to predict but, more importantly, to determine the factors that can be influenced to reduce the risk of infection. Among the above strong risk indicators affecting infection, consciousness disorder is mainly affected by the severity of the disease and is more difficult to treat, and CD4^+^ T cells can be used as candidate targets for intervention, so the mechanism underlying the decline in their levels needs more in-depth exploration.

After analyzing the results of the analysis of the subset of CSF lymphocytes in 11 patients with severe hemorrhagic stroke (Figure 4), CD4^+^ T-cell levels in CSF were found to increase significantly, and the CSF had significantly higher IL-6 and IL-8 levels than the blood; not only was a more obvious concentration gradient of chemotactic CD4^+^ T cells established, enabling them to enter the CSF, but IL-6 and IL-8 also increased the permeability of the blood–brain barrier (15, 23, 24), and it was speculated that a large number of CD4^+^ T cells in the blood were induced to enter the inflammatory site of the central nervous system (15, 25); this process resulted in a remarkable decrease of CD4^+^ T-cell levels in the blood, a decrease in the immunity of the body, and an increased risk for infection in patients. The migration of CD4^+^ T cells into the brain exacerbates neuroinflammation in the central nervous system and is involved in the secondary damage caused by hemorrhagic stroke (26, 27). Therefore, patients will probably benefit from methods that interfere with CD4^+^ T-cell migration into the central nervous system or increase peripheral blood CD4^+^ T-cell levels. In addition, it has been shown that hemorrhagic stroke causes synergistic activation of the hypothalamic–pituitary–adrenal axis and the sympathetic nervous system, resulting in the release of adrenal steroid hormones and catecholamines, causing a decrease in CD4^+^ T-cell levels in peripheral blood and immunosuppression (28).

Different lymphocyte subsets undertake different immune functions and are involved in inflammatory and immune processes in neurocritical diseases through cytokines and immunoglobulins (29–31). Detection of lymphocyte subsets is essential for the assessment and management of neurocritical patients.

The disadvantage of this study is that the samples were obtained from a single center and had certain limitations, further investigation or validation in a multicentre prospective clinical study is needed. However, the study was a worthwhile clinical exploration of infection in patients with severe hemorrhagic stroke.

In conclusion, we found that the decline in CD4^+^ T-cell levels in the blood after the onset of severe hemorrhagic stroke leads to an increased risk of early infection. CD4^+^ T-cell levels <300/μL in the blood can be used as threshold for the clinical prediction of early infection. The migration of CD4^+^ T cells from the blood to the CSF may be an intrinsic mechanism underlying their decline in the blood.

## Data availability statement

The raw data supporting the conclusions of this article will be made available by the authors, without undue reservation.

## Ethics statement

The studies involving human participants were reviewed and approved by the Ethics Committee of the First Affiliated Hospital of Zhengzhou University. Written informed consent from the participants' legal guardian/next of kin was not required to participate in this study in accordance with the national legislation and the institutional requirements.

## Author contributions

WM and JT conceived the study and supervised this work. YW performed data analysis and drafted the manuscript. JG, FY, RD, DS, PH, LW, GX, and SW assisted in collecting data. All authors reviewed and approved the final manuscript. All authors contributed to the article and approved the submitted version.
